# Single-Cell Genomics: Catalyst for Cell Fate Engineering

**DOI:** 10.3389/fbioe.2021.748942

**Published:** 2021-10-18

**Authors:** Boxun Li, Gary C. Hon

**Affiliations:** ^1^ Cecil H. and Ida Green Center for Reproductive Biology Sciences, University of Texas Southwestern Medical Center, Dallas, TX, United States; ^2^ Division of Basic Reproductive Biology Research, Department of Obstetrics and Gynecology, Department of Bioinformatics, University of Texas Southwestern Medical Center, Dallas, TX, United States

**Keywords:** reprogramming, single cell genomics, regenerative medicine, cell fate, transcription factor

## Abstract

As we near a complete catalog of mammalian cell types, the capability to engineer specific cell types on demand would transform biomedical research and regenerative medicine. However, the current pace of discovering new cell types far outstrips our ability to engineer them. One attractive strategy for cellular engineering is direct reprogramming, where induction of specific transcription factor (TF) cocktails orchestrates cell state transitions. Here, we review the foundational studies of TF-mediated reprogramming in the context of a general framework for cell fate engineering, which consists of: discovering new reprogramming cocktails, assessing engineered cells, and revealing molecular mechanisms. Traditional bulk reprogramming methods established a strong foundation for TF-mediated reprogramming, but were limited by their small scale and difficulty resolving cellular heterogeneity. Recently, single-cell technologies have overcome these challenges to rapidly accelerate progress in cell fate engineering. In the next decade, we anticipate that these tools will enable unprecedented control of cell state.

## Introduction

Progressive cell fate restriction is a central feature of organismal development famously illustrated by the “Waddington landscape” (Waddington, 1957). This model views cell fate establishment as irreversible. However, John Gurdon observed in *Xenopus* that nuclear transplantation of terminally differentiated cells into enucleated oocytes resulted in the development of normal frogs (Gurdon, 1962; Gurdon, 1967). This suggested that the nucleus does not permanently lose its potential to differentiate during development. In 1987, Davis and colleagues found that a gene specifically expressed in skeletal muscle, Myod1, converts mouse fibroblasts to skeletal muscle cells *in vitro* (Davis et al., 1987). In *Drosophila*, over-expression of the *eyeless* gene ectopically, eye structures are strikingly induced on the wings, the legs and the antennae (Halder et al., 1995). These studies clearly demonstrated the plasticity of terminally differentiated cells, and the possibility of engineering cell fate by gene over-expression. Two decades later, Takahashi and Yamanaka reprogrammed terminally differentiated cells to pluripotent stem cells with a cocktail of four transcription factors (TFs) (Takahashi and Yamanaka, 2006). This raised the important notion that cell fate engineering can be driven by a specific combination of TFs. Inspired by this breakthrough, many studies have extended this approach to reprogram pancreatic β-cells, cardiomyocytes, neurons, hepatocytes, and epicardial cells, among others ((Feng et al., 2008; Zhou et al., 2008; Ieda et al., 2010; Vierbuchen et al., 2010; Huang et al., 2011; Sekiya and Suzuki, 2011; Ladewig et al., 2012; Song et al., 2012; Nam et al., 2013; Niu et al., 2013; Batta et al., 2014; Chanda et al., 2014; Du et al., 2014; Riddell et al., 2014; Lemper et al., 2015; Duan et al., 2019) and reviewed in Xu et al. (2015), Wang et al. (2021).

This capability to engineer cell fate holds great promise in regenerative medicine, disease modeling, and drug discovery (Grath and Dai, 2019; Zhang et al., 2020). Two strategies are most commonly used for engineering cell fate: A) direct engineering (used interchangeably with direct reprogramming), defined as conversion of cell fate without passing through an intermediate pluripotent state), and B) differentiation from a pluripotent state, e.g., induced pluripotent stem cells. Both are viable approaches with important differences and unique advantages. The comparison between the two strategies is beyond the scope of this review, and are discussed elsewhere (Margariti et al., 2014; Cieślar-Pobuda et al., 2017). In this review, we focus on direct engineering, though many principles discussed here can applied to the differentiation strategy.

**FIGURE 1:**
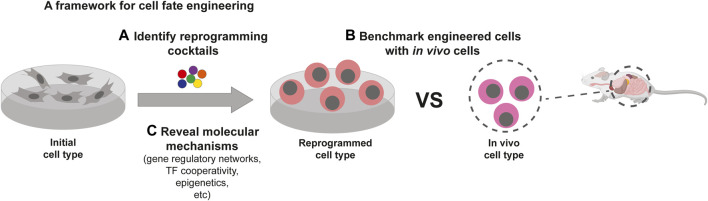
Three key pillars of cell fate engineering. A generalizable framework for cell fate engineering will require: **(A)** the ability to discover new reprogramming cocktails at scale, **(B)** reliable ways to benchmark engineered cells with their endogenous counterparts, and **(C)** a deeper understanding of the underlying molecular mechanisms of cell fate conversion. Part of this figure was created with BioRender.com.

Ideally, engineered cells need to faithfully recapitulate the target cell type at both molecular and functional levels. To extend cell fate engineering more broadly across cell types, tissues, and organisms, here we propose a methodological framework consisting of three pillars, based on current progress and future prospects of the field: 1) generalizable approaches to discovering new reprogramming cocktails at scale, 2) reliable ways to assess the engineered cells, benchmarked to their endogenous counterparts, and 3) comprehensive molecular mechanisms underlying cell fate engineering (Figure 1). Although stated separately, these areas of research are interrelated. For example, discovering new reprogramming cocktails usually involves some form of assessment of the engineered cells (Davis et al., 1987; Takahashi and Yamanaka, 2006; Zhou et al., 2008; Ieda et al., 2010; Vierbuchen et al., 2010; Song et al., 2012; Niu et al., 2013; Biddy et al., 2018; Duan et al., 2019); new molecular mechanisms often lead to improved reprogramming cocktails (Shu et al., 2013; Wapinski et al., 2013; Wang et al., 2015).

Despite recent progress, key challenges remain for each of the three pillars, which we will review here. First, we review the rationale and challenges of traditional methods that were developed for direct cell fate engineering before the advent of single-cell genomics. Second, we discuss promising single-cell genomic approaches that have emerged to address some of these challenges. Finally, we discuss the promise of applying this framework to *in vivo* reprogramming. Overall, we anticipate that single-cell approaches will play a key role in establishing a generalizable framework for cell fate engineering.

### Traditional Methods: Rationale and Challenges

#### Pillar 1: Discovering Reprogramming Cocktails

Discovering cocktails of reprogramming factors is a two-step process. First, candidate genes must be selected. Several criteria are commonly used to identify candidate TFs that: 1) play a role in the natural development of target cell type, 2) manifest a relevant developmental phenotype when knocked out, and/or 3) are specifically expressed in the target cell type (Takahashi and Yamanaka, 2006; Ieda et al., 2010; Vierbuchen et al., 2010; Song et al., 2012). This curation step reduces the number of candidate genes to functionally test, which is critical to reduce the combinatorial space searched. However, these criteria also constrain these experiments to well-studied cell types.

Second, candidate genes are narrowed down to identify the smallest cocktail that efficiently reprograms cell fate. To efficiently achieve this goal, traditional approaches include the use of engineered reporters for successful cell fate conversion. Many studies rely on a single endogenous gene reporter that is engineered to be specifically expressed in the target cell type (Takahashi and Yamanaka, 2006; Ieda et al., 2010; Vierbuchen et al., 2010; Song et al., 2012). This is a useful way to simplify the readout and reduce the workload of screening through many cocktails. However, this convenience comes at the cost of two disadvantages: 1) a single reporter gene of successful reprogramming may not be known a priori or may not exist, and 2) the reporter may require the time-consuming task of genetically engineering cells or organisms. Another traditional approach used to increase efficiency, since the entire combinatorial space among all candidate genes is too prohibitive to search, is a ‘minus one’ experimental strategy that iteratively tests each factor’s role in reprogramming by removing it from the pool (Takahashi and Yamanaka, 2006; Ieda et al., 2010; Vierbuchen et al., 2010; Song et al., 2012). If the removal of a factor does not reduce or even increases the reprogramming efficiency, then it is deemed unnecessary and excluded from the pool. This is repeated multiple times until no factor can be subtracted without compromising reprogramming efficiency. This approach has two disadvantages. First, it is labor-intensive and tedious, making it hard to scale up to multiple target cell types. Second, this approach only searches a small proportion of the full combinatorial space among all candidate genes. This raises the possibility of missing alternative or more efficient cocktails. Indeed, Hand2 was shown to enhance the efficiency of the original GMT (Gata4, Mef2c, Tbx5) cocktail for cardiomyocyte reprogramming (Song et al., 2012). Intriguingly, in the screen conducted by Ieda and colleagues, removal of Hand2 increased reprogramming efficiency, and was thereby excluded from the cocktail (Ieda et al., 2010). This suggests that important TF interactions in reprogramming might not be readily revealed by the traditional screening approach.

Recent genomic strategies have addressed several shortcomings of traditional approaches. Two groups used CRISPR gene activation technology to screen through large numbers of putative TFs and other DNA-binding factors (2,428 and 1,496, respectively) for neuronal fate specification ability (Liu et al., 2018; Black et al., 2020). These scales are impressive, approximating the total number of all putative TFs in the human genome (Lambert et al., 2018). However, the studies have two limitations. First, they still rely on an endogenous reporter gene, the drawbacks of which have been discussed above. Second, since the screens were conducted in bulk, combinatorial perturbation information is lost. However, interaction within reprogramming cocktails is critical for cell fate engineering. For example, two studies screening hundreds of TF pairs for neuronal reprogramming performance revealed prevalent synergies between TFs that enhance reprogramming (Liu et al., 2018; Tsunemoto et al., 2018). While these studies highlight the importance of TF interactions, large scale screening of combinatorial TF cocktails remains challenging.

Recently, a suite of computational approaches has been developed to predict the reprogramming abilities of TFs and prioritize candidate TF cocktails to test experimentally (Cahan et al., 2014; Morris et al., 2014; D’Alessio et al., 2015; Rackham et al., 2016; Jung et al., 2021). Many of these methods rely on gene regulatory networks (GRNs) that link TFs to their target genes. These GRNs are often constructed from bulk gene expression datasets from diverse cell types and tissues, sometimes supplemented by bulk epigenetic data (Jung et al., 2021). These methods greatly reduce the combinatorial space of TF cocktails that need to be tested experimentally, addressing a major challenge posed above. Indeed, these methods have shown promising success in improving current reprogramming cocktails (Cahan et al., 2014; Morris et al., 2014), and predicting known (Rackham et al., 2016; Jung et al., 2021) and new (D’Alessio et al., 2015; Jung et al., 2021) reprogramming TFs. However, it remains to be seen if these methods are generalizable to a large number of target cell types. Moreover, one common drawback of these methods is that they often only use bulk, but not single-cell, expression and epigenetic datasets. As discussed more in detail below, bulk assays average across multiple cell types that often coexist in a given tissue, adding noise to cell type-specific GRN reconstruction that is key to the predictive power of these computational methods. As a result, future iterations of these methods should take advantage of the fast-expanding single-cell atlases to resolve the heterogeneity of bulk samples.

In summary, traditional strategies to discover reprogramming cocktails have advantages and disadvantages. Notably, the disadvantages stem from the lack of large-scale combinatorial screens, which limits both the scale and exhaustiveness of cocktail discovery. New technologies will be needed to address this challenge.

#### Pillar 2: Assessing Engineered Cells

Evaluating how well engineered cells recapitulate the molecular and functional features of endogenous cells is a critical task.

**FIGURE 2:**
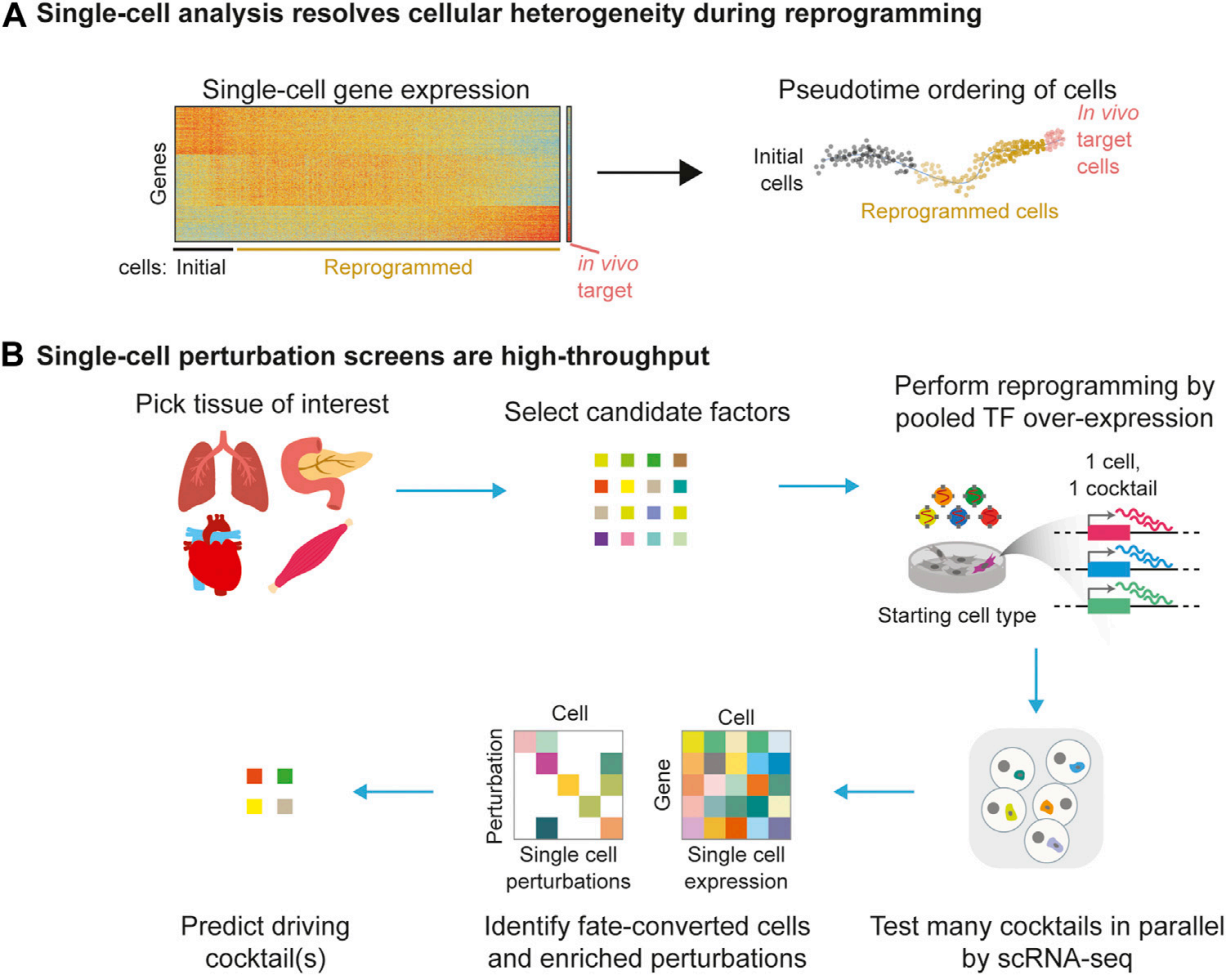
Applications of single-cell genomics to cell fate engineering. **(A)** Single-cell analysis of reprogramming resolves cellular heterogeneity and reprogramming asynchrony. **(B)** Single-cell perturbation screens can scale the testing of reprogramming cocktails. Each sequenced cell tests a specific cocktail, and sequencing thousands of cells allows many cocktails to be simultaneously tested in one experiment.

Molecular approaches are generalizable to different target cell types. Methods with simple readouts, such as real-time quantitative PCR and immunofluorescence, interrogate the changes of individual marker gene expression, usually targeted against well-established specific marker genes for the target cell type (Davis et al., 1987; Takahashi and Yamanaka, 2006; Zhou et al., 2008; Ieda et al., 2010; Vierbuchen et al., 2010; Song et al., 2012; Niu et al., 2013; Duan et al., 2019). While they are easy to implement, the expression of a handful of marker genes is hardly sufficient evidence of cell fate conversion. Thus, genome-scale readouts are often used to measure global changes in RNA or epigenetic (DNA methylation, chromatin accessibility, and histone marks) status in engineered cells (Takahashi and Yamanaka, 2006; Ieda et al., 2010; Wapinski et al., 2013; Cahan et al., 2014; Morris et al., 2014; Liu et al., 2016; Wapinski et al., 2017; Stone et al., 2019). These comprehensive molecular analyses have generated important insights. For example, by using bulk RNA microarray and ChIP-seq data to benchmark engineered cells across several target cell types, the CellNet studies illustrated that virtually all reprogramming paradigms fail to completely silence the gene expression programs of the starting cell (Cahan et al., 2014; Morris et al., 2014). While powerful, bulk genomic methods share a fundamental limitation: they take an average measurement of all cells in a population, thereby missing the heterogeneity and asynchrony of cell fate engineering (Treutlein et al., 2016; Liu et al., 2017; Biddy et al., 2018; Schiebinger et al., 2019; Zhou et al., 2019) (Figure 2A).

Functional assessment of engineered cells is vital for applications in regenerative medicine and disease modeling. This step entails testing the cellular functions most characteristic of and fundamental to the target cell type (Takahashi and Yamanaka, 2006; Zhou et al., 2008; Ieda et al., 2010; Vierbuchen et al., 2010; Song et al., 2012; Niu et al., 2013). For example, induced pluripotent stem cells are tested for their ability to differentiate into all three germ layers, as well as their contribution to mouse embryonic development (Takahashi and Yamanaka, 2006); induced pancreatic β-cells, their ability to secrete insulin and regulate blood glucose level (Zhou et al., 2008); induced cardiomyocytes, spontaneous beating and intracellular Ca^2+^ flux (Ieda et al., 2010). Furthermore, engineered cells need to survive engraftment and integrate with local cells *in vivo* (Ieda et al., 2010), if they are to be used for regenerative medicine. However, given that each target cell type has unique functions, functional assays are necessarily selected on an ad hoc basis, making it hard to generalize. As such, they are better reserved as the most stringent test, ideally performed after the cells pass the molecular tests mentioned above.

#### Pillar 3: Molecular Mechanisms

Here, we discuss two important molecular mechanisms of reprogramming: the combinatorial interactions of reprogramming TFs and the role of the epigenome.

Reprogramming cocktails usually contain multiple TFs, and many exhibit cooperative roles in reprogramming. First, some reprogramming TFs have pioneering activity (Cirillo et al., 2002; Soufi et al., 2012; Buganim et al., 2013; Wapinski et al., 2013), such as Gata4 in cardiomyocyte reprogramming (Ieda et al., 2010; Song et al., 2012), and Ascl1 in neuronal reprogramming (Vierbuchen et al., 2010). Pioneering is defined as the ability to bind regions of closed chromatin (Cirillo et al., 2002; Wapinski et al., 2013). It has been posited that pioneering TFs are essential to initiating cell fate engineering, but the maturation of product cells requires assistance from maturation factors, which is why the vast majority of reprogramming cocktails consists of at least one pioneering factor, plus other non-pioneering factors (Morris, 2016). Second, reprogramming TFs often interact to yield joint activities that are beyond individual TFs. For example, in neuronal reprogramming, Ascl1 binding recruits Brn2 to sites which are inaccessible to Brn2 alone (Wapinski et al., 2013). In cardiomyocyte reprogramming, Gata4, Mef2c, and Tbx5 cooperatively bind at cardiomyocyte-related genomic regions, and refine each other’s binding affinity to certain regions when co-expressed (Stone et al., 2019). Moreover, Hand2 and Akt1 enhance the co-occupancy of GMT and GHMT at cardiomyocyte-related developmental enhancers, respectively (Hashimoto et al., 2019). Finally, the doses of TFs within a cocktail are important, shown by an elegant study using polycistronic constructs which found that GMT reprogramming is the most efficient when Mef2c is expressed more than the other two factors (Wang et al., 2015). This concept of a balance between reprogramming factors is further demonstrated by a seesaw model that argues that OCT4 and SOX2 counteract each other to achieve pluripotent reprogramming (Shu et al., 2013), among other reports that factor stoichiometry affects iPSC reprogramming efficiency and quality (Papapetrou et al., 2009; Carey et al., 2011; Tiemann et al., 2011; Yamaguchi et al., 2011). To sum up, many important insights have been gained into the molecular mechanisms of reprogramming factors. With the discovery of more and more reprogramming cocktails, an important goal for future studies will be to determine the universality of these mechanisms to derive general rules of reprogramming.

Cell fate conversion requires both transcriptional (Takahashi and Yamanaka, 2006; Ieda et al., 2010; Cahan et al., 2014; Morris et al., 2014) and epigenetic reprogramming (Liu et al., 2016; Wapinski et al., 2017; Luo et al., 2019; Stone et al., 2019). Transcriptionally, reprogrammed cells activate target cell type-specific gene programs, and silence those from initial cell states (Takahashi and Yamanaka, 2006; Ieda et al., 2010). Yet, conversion often remains incomplete (Cahan et al., 2014; Morris et al., 2014). Epigenetically, chromatin accessibility, histone marks, and DNA methylation are globally reprogrammed (Liu et al., 2016; Wapinski et al., 2017; Luo et al., 2019; Stone et al., 2019). These are pivotal observations. However, one common drawback of the methods used in these studies is that they have most frequently been applied to bulk samples, which ignores the heterogeneity and asynchrony of reprogramming. For example, is incomplete transcriptional conversion (Cahan et al., 2014; Morris et al., 2014) a result of all cells being incompletely converted, or a mixture of fully and incompletely reprogrammed cells? Such questions can only be answered by single-cell analysis.

In summary, it is crucial to understand the functions and interaction of reprogramming factors, yet such knowledge is difficult to generate at large scale. Furthermore, our understanding of transcriptional and epigenetic changes that underlie cell fate engineering is limited by our ability to resolve heterogeneity in reprogramming. Single-cell approaches hold promise in addressing these gaps in knowledge.

### Single-Cell Technologies: Promise and Limitations

Among the challenges discussed above, two themes are prominent and recurrent in traditional bulk reprogramming: resolution and scale (Figure 2). On one hand, bulk approaches cannot resolve the well-known heterogeneity in cell fate engineering. This hampers both the assessment of product cells and molecular mechanisms. On the other hand, bulk approaches also reduce the scale at which reprogramming cocktails can be tested. This limits the speed at which we can discover new cocktails and investigate molecular mechanisms. Excitingly, the advent of single-cell technologies have shown great promise in addressing these two challenges, albeit with their own limitations. Here, we discuss single-cell technologies with respect to the challenges of resolution and scale in cell fate engineering.

#### Single-Cell Omics Resolves Heterogeneity in Reprogramming

In the past decade, single-cell technologies have made great strides in lowering cost, increasing scale, and enabling new readouts (genomics, transcriptomics, and epigenomics) at unprecedented resolution (Telenius et al., 1992; Spits et al., 2006; Hashimshony et al., 2012; Ramsköld et al., 2012; Zong et al., 2012; Guo et al., 2013; Nagano et al., 2013; Picelli et al., 2013; Sasagawa et al., 2013; Smallwood et al., 2014; Buenrostro et al., 2015; Macosko et al., 2015; Rotem et al., 2015; Cao et al., 2017; Gierahn et al., 2017; Habib et al., 2017; Zheng et al., 2017; Han et al., 2018; Kaya-Okur et al., 2019; Ku et al., 2019), and reviewed in Kashima et al. (2020). These advances improve the assessment of engineered cells and aid the revelation of new molecular mechanisms by resolving heterogeneity (Figure 2A).

Single-cell RNA-sequencing (scRNA-seq) (Hashimshony et al., 2012; Ramsköld et al., 2012; Picelli et al., 2013; Sasagawa et al., 2013; Macosko et al., 2015; Cao et al., 2017; Gierahn et al., 2017; Habib et al., 2017; Zheng et al., 2017; Han et al., 2018) has been widely adopted in reprogramming studies and has led to several key observations. First, an early study of iPSC reprogramming analyzed 48 select genes of single cells during reprogramming (Buganim et al., 2012). The authors made an important observation that reprogramming is heterogeneous, with first a “stochastic” phase and then a more “deterministic” phase with Sox2 as the master regulator. This helps explain why only a small fraction of cells reach an iPSC fate. Second, a transcriptome-wide single-cell analysis of neuronal reprogramming induced by Ascl1, Brn2, and Myt1l strikingly revealed a reprogramming trajectory in which Ascl1 not only activates neuronal, but also myocytic, genes in fibroblasts, the latter of which are repressed by Brn2 and Myt1l (Treutlein et al., 2016). Therefore, cells that fail reprogramming might end up in an unproductive “dead-end” branch. Thirdly, a scRNA-seq analysis of human cardiomyocyte reprogramming induced by GMT revealed a decision point at which fibroblasts either progress further and become fully converted, or revert back to the fibroblast fate (Zhou et al., 2019). This raises the possibility that the previous observation of “incomplete” reprogramming (Cahan et al., 2014; Morris et al., 2014) is due to a mixture of successful and failed cells. Fourthly, scRNA-seq with dense time point sampling during iPSC reprogramming (Schiebinger et al., 2019) showed that “off target” cell fates are adopted, including stromal, trophoblast-like, and neuronal fates. Furthermore, the authors developed a computational method, Waddington-OT, that reconstructs the reprogramming trajectory, revealing a thin bottleneck for iPSC reprogramming. This conclusion was made possible because of the dense time points sampled, and demonstrates the power of using improved experimental designs to empower analytical methods. Moreover, they identified an environmental cue, GDF9, that is secreted by the stromal lineage to facilitate iPSC lineage reprogramming, echoing observations elsewhere that microenvironment plays an important role in reprogramming ((Mosteiro et al., 2016), also reviewed in Wang and Zhang (2018)). Finally, paralleling efforts on direct reprogramming, single-cell perturbation screens have also been applied to models of differentiation to resolve the functions of transcription factors during heterogeneous cell fate changes. Studies in definitive endoderm and teratoma differentiation delineate the TFs necessary for each cell state transition (Genga et al., 2019; McDonald et al., 2020).

Aside from the transcriptome, the measurement of other cellular features at single-cell resolution is still relatively immature. Nevertheless, some techniques have already been applied to cell fate engineering. First, single-cell transcriptomics analysis combined with lineage tracing of fibroblast to endoderm progenitor reprogramming (Biddy et al., 2018) identified a successful path and a dead-end path. Interestingly, the cells commit to a path very early in reprogramming, when their global gene expression patterns have not yet diverged. Second, leveraging both scRNA-seq and single-cell ATAC-seq data, the same group developed CellOracle, a computational method to reconstruct GRNs which enabled them to identify additional factors at play in endoderm progenitor reprogramming (Kamimoto et al., 2020). Finally, with the development of new single-cell technologies that jointly measure multiple features in the same cell (Angermueller et al., 2016; Peterson et al., 2017; Stoeckius et al., 2017; Chen et al., 2019; Liu et al., 2019; Rooijers et al., 2019) and also reviewed in Kashima et al. (2020)), we anticipate that the transcriptomic and epigenomic changes in cell fate engineering will be increasingly dissected at the single-cell level, yielding broader and deeper insights.

Several limitations of single-cell technologies are worth noting. First, we and others observed that even at single-cell level, the most successfully reprogrammed cells are still not equivalent to their endogenous counterparts (Shin et al., 2012; Duan et al., 2019), at least at the transcriptional level. This could be due to incomplete maturation of product cells, the lack of the favorable microenvironment of the endogenous cells, or *in vitro* culture conditions. Second, transcriptional reprogramming does not necessarily dictate cellular functions. Therefore, functional validation is critical to complement single-cell omics assessment. Third, single-cell data is noisy (Adil et al., 2021), and the parameters of computational methods can influence data interpretation. Care should be taken to ensure that conclusions are robust across multiple parameters and algorithms.

#### Large-Scale Perturbation Screens Accelerate Cocktail Testing

Single-cell perturbation screens link each cell’s transcriptome to its perturbation identity (Adamson et al., 2016; Dixit et al., 2016; Jaitin et al., 2016; Datlinger et al., 2017; Xie et al., 2017; Gasperini et al., 2019; Xie et al., 2019; Luginbühl et al., 2021). This methodology is powerful because it treats every sequenced cell as an independent experiment, thus measuring the transcriptional impact of genetic perturbations at scales previously unimaginable. For example, thousands of perturbations have been assayed in a recent study (Gasperini et al., 2019). In these screens, common types of perturbation are gene knockouts facilitated by genome editing (Dixit et al., 2016; Jaitin et al., 2016; Datlinger et al., 2017), gene knockdown by epigenome editing (i.e., CRISPR interference) (Adamson et al., 2016; Xie et al., 2017; Gasperini et al., 2019; Xie et al., 2019), and open reading frame over-expression of TFs (Parekh et al., 2018; Duan et al., 2019; Luginbühl et al., 2021). In the context of reprogramming, open reading frame over-expression is the most common mode of perturbation, used in all three single-cell perturbation studies (Parekh et al., 2018; Duan et al., 2019; Luginbühl et al., 2021), though gene activation and knockdown have also been used to achieve reprogramming (Liu et al., 2018; Qian et al., 2020; Zhou et al., 2020).

Before high-throughput scRNA-seq was widely available, an early study demonstrated the feasibility of screening for monocyte reprogramming factors at the single-cell level using multiplexed single-cell qPCR (Shin et al., 2012). Since only dozens of genes can be simultaneously measured in this way, the study required the assistance of the enrichment of established cell type-specific surface markers to report cell fate conversion. Applying modern single-cell perturbation screens to cell fate engineering, we and others have over-expressed pools of candidate factors and measured the joint readout of transcriptome and perturbation (Parekh et al., 2018; Duan et al., 2019; Luginbühl et al., 2021) of single cells (Figure 2B). This approach utilizes the transcriptomic readout to identify successfully reprogrammed cells, without relying on reporter genes. Then, the perturbation readout can be used to identify drivers of reprogramming. By undirected and directed combinatorial screening of 48 factors and 10 factors, respectively, we discovered Atf3, Gata6, and Hand2 to be a reprogramming cocktail for epicardial cells (Duan et al., 2019). Luginbühl and colleagues similarly identified previously unknown cocktails among 20 pro-neuronal TFs to engineer different neuronal subtypes (Luginbühl et al., 2021). Applying this concept to pluripotent stem cell differentiation models, Parekh and colleagues over-expressed 61 TFs and identified ETV2 as an inducer of endothelial fate (Parekh et al., 2018). These approaches are poised for expansion to more cell types. If successful, they will accelerate the discovery of new reprogramming cocktails.

Besides over-expression screens, repression screens of potential barriers of cell fate conversion are also of tremendous interest (Dhawan et al., 2011; Courtney et al., 2013; Tomaru et al., 2014; Zhou et al., 2016; Qian et al., 2020), also reviewed in Jenuwein and Allis (2001), Xu et al. (2015), Wang et al. (2021)). For example, the inhibition of epigenetic modifiers, such as histone deacetylases and polycomb complexes, and an RNA-binding protein, PTB, have been shown to facilitate or induce reprogramming (Xu et al., 2015; Zhou et al., 2016; Qian et al., 2020). In addition, the knock-down of four core regulators of dermal fibroblast cell fate enables adipogenesis under induction medium (Tomaru et al., 2014), suggesting that reprogramming can further benefit from disrupting combinations of factors in the starting cell type. In *c. elegans*, chromatin-regulating proteins including LIN-53, FACT, and MRG-1 have been shown to safeguard cell fate (Tursun et al., 2011; Kolundzic et al., 2018; Hajduskova et al., 2019). Knocking down these proteins facilitates cell fate conversion. Taken together, applying single-cell knock-down screens to existing master regulators or guardians of the starting cell fate could yield important insights into cell fate maintenance as well as powerful ways to facilitate reprogramming.

Single-cell combinatorial analysis of TFs can also yield new insights on molecular mechanisms. For example, this analysis can generate functional interaction data at large scale. Since single-cell combinatorial perturbation screens measure both the transcriptome and TF perturbation of a cell, single cells could be grouped computationally based on their perturbation. By comparing the transcriptional effect of two single TFs, A and B, and that of both TFs together, AB, the interaction between A and B can be measured and modeled by established methods for analyzing genetic interactions, as described by Norman and colleagues (Norman et al., 2019). These mechanistic insights could be informative for the design of reprogramming cocktails.

### *In Vivo* Cell Fate Engineering: Advantages and Challenges

*In vivo* cell fate engineering induces reprogramming factors *in vivo* (Zhou et al., 2008; Song et al., 2012; Niu et al., 2013), and presents unique advantages and challenges compared to *in vitro* reprogramming.

There are two important advantages. First, the *in vivo* microenvironment might be conducive to cell fate engineering, resulting in more efficient reprogramming and more mature product cells. For example, the three-factor cocktail that reprograms pancreatic exocrine cells to β-cells only works *in vivo*, but not *in vitro* (Zhou et al., 2008); cardiomyocyte reprogramming by GMT is more efficient *in vivo* than *in vitro* (Qian et al., 2012). Furthermore, neural injury and degeneration/aging have a positive impact on reprogramming (reviewed in Wang and Zhang (2018)). Second, *in vivo* cell fate engineering circumvents genetic mutations induced by *in vitro* culture, eliminating a major risk for regenerative medicine (Taguchi and Yamada, 2017).

However, there are also challenges associated with applying the methodological framework for cell fate engineering *in vivo*. First, *in vitro* findings of reprogramming cocktails do not always extrapolate to *in vivo* conditions ((Zhou et al., 2008), and reviewed in Tai et al. (2020)). Therefore, to achieve *in vivo* reprogramming, directly screening for cocktails *in vivo* is ideal. However, combinatorial gain-of-function screens *in vivo* are challenging to perform. As a result, *in vivo* gain-of-function TF screens have been limited to small numbers of candidate factors (Zhou et al., 2008; Niu et al., 2013). Therefore, there is an urgent need for scalable screening methods. Second, reprogrammed cells *in vivo* are embedded with endogenous cells, making it critical to distinguish engineered from non-engineered cells. Distinguishing these two kinds of cells is crucial to assess product cells and reveal molecular mechanisms. As such, extensive lineage tracing experiments are usually carried out in such studies (Zhou et al., 2008; Qian et al., 2012; Song et al., 2012; Grande et al., 2013; Niu et al., 2013; Guo et al., 2014).

## Concluding Remarks

The field of cell fate engineering has made tremendous progress in the past 2 decades. In the 2000s, traditional bulk reprogramming approaches established a strong foundation for TF-mediated reprogramming, but were limited by cellular heterogeneity and small scale. In the 2010s, the development and application of single-cell technologies to cellular reprogramming has accelerated recent progress. In the coming decade, we anticipate that single-cell technologies will revolutionize the discovery of new reprogramming cocktails, the evaluation of engineered cells, and the molecular mechanisms of cell fate.
